# A new model for learning-based forecasting procedure by combining k-means clustering and time series forecasting algorithms

**DOI:** 10.7717/peerj-cs.534

**Published:** 2021-06-02

**Authors:** Kristoko Dwi Hartomo, Yessica Nataliani

**Affiliations:** Department of Information System, Faculty of Information Technology, Satya Wacana Christian University, Salatiga, Central of Java, Indonesia

**Keywords:** Forecasting, Clustering, k-means, Learning-based

## Abstract

This paper aims to propose a new model for time series forecasting that combines forecasting with clustering algorithm. It introduces a new scheme to improve the forecasting results by grouping the time series data using k-means clustering algorithm. It utilizes the clustering result to get the forecasting data. There are usually some user-defined parameters affecting the forecasting results, therefore, a learning-based procedure is proposed to estimate the parameters that will be used for forecasting. This parameter value is computed in the algorithm simultaneously. The result of the experiment compared to other forecasting algorithms demonstrates good results for the proposed model. It has the smallest mean squared error of 13,007.91 and the average improvement rate of 19.83%.

## Introduction

Currently, climate change affects rainfall patterns. The negative impact of changes in rainfall patterns is the occurrence of extreme floods and droughts (Hecht, 2016; Mislan et al., 2015; Strategy, 2011). Rainfall forecast information is an important requirement to support water resource management and anticipation of disasters, especially when climate change occurs (Mislan et al., 2015). Forecasting using a time series model basis aims to study previous observations based on the collected data and build a suitable forecasting model (Naim, Mahara & Idrisi, 2018). Previous studies on time series data forecasting show that the errors of forecasting are still significant and the forecasting is still inaccurate to predict rainfalls and weather. One of the reasons is because the weather data have a non-linear structure (Haviluddin & Alfred, 2014; Shrivastava et al., 2012). However, in another study, the statistical methods of rainfall forecasting have been able to produce accurate forecasts (Farajzadeh, Fard & Lotfi, 2014). Rainfall forecasting with a good and accurate method is needed to anticipate the negative impact of extreme weather (Manton et al., 2001; Yusuf & Francisco, 2017). The lack of knowledge about the future, and the term projections, whether it is short, medium or long term, make forecasting methods indispensable in planning, management, and anticipation of arising the negative impacts (Dantas, 2018). Forecasting methods that can accurately predict the future will have a significant contribution to calculate uncertainty. It allows a more efficient decision making (Hyndman & Athanasopoulos, 2014). For decades, there have been many efforts to obtain an accurate forecasting result. Researchers have also developed statistical models and forecasting methods (De Goojier, Hyndman, 2006).

The exponential smoothing algorithm is a short-term method and it is often called an inconsistent forecasting method. One example would be the case of the decrease in agricultural production in an area caused by drought. However, this exponential smoothing model will still describe an increase in its production (Burkom, Murphy & Shmueli, 2007; Hyndman et al., 2002). Forecasting using a smoothing algorithm is only effective for short term (Hameed, 2015; Ngopya, 2009). In the exponential smoothing method, the important parameter is the smoothing constant (*α*) representing the percentage of estimating error (Karmaker, 2017). The main weakness of this method is the process of determining the optimal smoothing constant. The evaluation of forecasting accuracy depends on the smoothing constant value. The optimal value of the constant is processed using the lowest mean absolute error, mean absolute percentage error, and root mean squared error (Karmaker, 2017; Khairina et al., 2019; Ostertagová & Ostertag, 2013). In order to determine the optimal exponential smoothing value with minimum error, forecasting is made through a trial and error method (Hameed, 2015; Karmaker, 2017; Paul, 2011). Determination of a smoothing constant through a trial and error method is considered as an ineffective method. Unsuitable smoothing constant will give inaccurate forecast result. The experimental results in previous studies indicate that a single exponential smoothing is not suitable for predicting data with trending cases or seasonal time series (Green & Armstrong, 2015; Kourentzes, Rostami-Tabar & Barrow, 2017; Lim, 2011; Prema & Rao, 2015).

The exponential smoothing method is a very successful forecasting method and widely used in theoretical research (Maia & de Carvalho, 2011; Chen & Seneviratna, 2014; Jose & Winkler, 2008; Kolassa, 2011; Kourentzes, Petropoulos & Trapero, 2014). The conducted research focuses on improving the performance and accuracy of exponential smoothing forecasting method, especially the single exponential smoothing. The proposed new model is based on the single exponential smoothing because it is a simple forecasting method that requires only small sample data and has a comprehensive statistical framework for short-term forecasting (Khairina et al., 2019; Hyndman et al., 2002; Zhao, Mbachu & Zhang, 2019). M-Competition found that the simplest extrapolation method which is suitable for time series data forecasting is the single exponential smoothing. Its forecasting accuracy is close to 16 more complex forecasting methods (Gardner & Diaz-Saiz, 2008; Green & Armstrong, 2015). Empirical study shows that forecasting with complex and sophisticated statistical methods might be less accurate than forecasting using simple methods (Lee, Song & Mjelde, 2008).

Recently, machine learning has become popular in the world driven by the advancement and development of computers that have made high performance servers available at low cost (Dantas & Oliveira, 2018). One part of machine learning is clustering the unsupervised learning technique category (Haraty, Dimishkieh & Masud, 2015; Nataliani & Yang, 2019; Patel & Mehta, 2011). Unlike classification, clustering is a type of unsupervised learning with unlabeled data, in which the number of class is not used in the method of grouping (Haraty, Dimishkieh & Masud, 2015). In clustering, large data sets are partitioned into smaller subgroups or groups based on their similarity measures (Kulis & Jordan, 2012). This approach is mainly applied to find similarities between data points. One of the clustering methods that is suitable to use in time series is k-means (Huang et al., 2016; Liao, 2005). The k-means method is suitable for the pre-processing time series data, in which the datasets then will be grouped. Finished with that grouping, the outlier data, the inconsistent data and noise data are removed, which then resulted in only the valid data from the pre-processing stage are forecasted (Santhanam & Padmavathi, 2015). The k-means clustering algorithm is used because of the efficient nature, wide scalability, and simplicity in the process. Besides, this algorithm yields better accuracy than hierarchical clustering algorithm (Riyadi et al., 2017; Shete & Buchade, 2019).

Forecasting techniques that combine classical statistical models and machine learning is gaining popularity in the research and literature studies for time series forecasting. Recent studies on the accuracy of forecasting results with those techniques has shown promising results (Bergmeir, Hyndman & Benítez, 2016; Štěpnička & Burda, 2017).

The paper aims to improve the forecasting accuracy of one of the forecasting methods, i.e., single exponential smoothing. A modified single exponential smoothing, named learning-based single exponential smoothing algorithm (LSES) will be proposed. The first step is combining the time series forecasting method with unsupervised learning technique, i.e., k-means clustering algorithm. The second step is to create a new procedure to calculate smoothing constant (alpha) using learning-based method to find the most optimal smoothing value. Previous studies determine smoothing constant through trial and error processes. To evaluate the performance of LSES, the proposed method is compared to five leading algorithms: single exponential smoothing, double exponential smoothing, triple exponential smoothing, and auto Arima, and exponential smoothing seasonal planting index (ESSPI). Experimental results and comparisons show that LSES algorithm produces better forecasting results.

This paper is presented as follows. ‘Introduction’ provides the background research on some weaknesses and advantages of the single exponential smoothing and the k-means methods. From this background, it then proposes a new method of time series data forecasting based on the single exponential smoothing that has a better accuracy than that of other forecasting methods. ‘Literature review’ briefly describes the reviews of the single exponential smoothing and k-means methods in the literature study. ‘The proposed method: learning-based single exponential smoothing algorithm’ presents the research method and flowchart, as well as the proposed algorithm. This study proposes a new method of time series data forecasting by combining the single exponential smoothing method and the k-means clustering method, called learning-based single exponential smoothing (LSES) to improve the accuracy of forecasting. The novelty and contribution include creating a new procedure to calculate the smoothing constant (alpha) in the single exponential smoothing based on learning method to find the optimal smoothing value. ‘Experimental results’ explains the experiments and comparisons of the proposed method with other time series data forecasting methods using real rainfall data. The graphs are used to present comparisons and analysis of the results of forecasting experiments. Finally, the conclusions are stated in ‘Conclusions’.

The contributions of the paper can be summarized as follows.

• It proposes a new scheme to improve the forecasting method by clustering the data and utilize that clustering result to forecast the data.
• It proposes a learning procedure for estimating the smoothing coefficient that will be used needed on the forecasting method. This smoothing coefficient is computed in the algorithm, simultaneously.

## Literature Review

In this section, some related works are presented. Below are the abbreviations and notations used in this paper:

• ARIMA: autoregressive integrated moving average
• GM: grey model
• LV: Lotka-Votterra
• SES: single exponential smoothing
• DES: double exponential smoothing
• TES: triple exponential smoothing
• ESSPI: exponential smoothing seasonal planting index
• LSES: learning based single exponential smoothing
• MSE: mean squared error
• MAE: mean absolute error
• MAD: mean absolute deviation
• MAPE: mean absolute percentage error
• MASE: mean absolute scaled error
• MSD: mean squared deviation
• $X= \left\{ {x}_{1},\ldots ,{x}_{n} \right\} $ is the data, where *x*_i_ is the *i*th data and *n* is the number of data
• *V* = *v*_1_, …, *v*_*c*_ is the cluster center, where *v*_k_ is the *k*th cluster center and *c* is the number of cluster
• $Z={ \left[ {z}_{ik} \right] }_{n\times c}$, where *z*_*ik*_ is the membership partition of the *i*th data in the *k*th cluster
• *X*_*t*_: the actual data in period *t*
• *F*_*t*_: the forecast data in period *t*
• *α*_*k*_: the smoothing value parameter of the *k*th cluster
• *W*_*k*_: the clustered data
• ${\hat {W}}_{k}$: the normalized clustered data

Hyndman et al. (2002) proposed a new approach to perform an automatic forecasting based on various exponential smoothing methods. The results of automatic forecasting using M-Competition data and IJF-M3 competition data show a good forecasting accuracy for short-term prediction intervals (up to about six periods ahead) - YEAR 2002 (Hyndman et al., 2002). Subsequent research was carried out on the background of the importance of efficient study of temporal rainfall pattern in hydrological management. They explain that their study was carried out across the country to model a rainfall trend in Pakistan over the past six decades. For this purpose, the secondary dataset of average rainfall for 65 years was made for the period 1951 to 2015. In Pakistan, adverse consequences of rainfall had been observed, which were in the form of drought and flash floods that had a devastating effect on human settlements, water management, and agriculture. In this study, data were analyzed using a sliced functional time series model, which was a relatively new for forecasting method. The results showed a downward trend in the average rainfall across the country. The monthly forecast for the next ten years (2016–2025) was obtained along with a prediction interval of 80%. This forecast was also compared with the forecast obtained from the ARIMA model and exponential smoothing state space (ETS) (Yasmeen & Hameed, 2018).

Subsequent research was carried out concerning the time series data forecasting using the single exponential smoothing method with the error measurement methods of MAPE, MAD, and MSE. Researchers conducted nine trials to determine the most optimal smoothing constant (*α*), in which the test results showed that the greater of smoothing constant value gave a better forecasting accuracy. The values of MAPE, MAD, and MSE decreased along with increasing smoothing constant value. Research showed that minimum error occurred at constant optimal smoothing (*α* = 0.9) which resulted in MAPE of 13.1, MAD of 117.4, and MSD of 26,912.1 (Karmaker, Halder & Sarker, 2017).

Another research has compared the ability of three forecasting models using limited historical data. Based on monthly data on tourist arrivals for the period 2001 to 2013, three simple forecasting models that did not require many historical data were used for model construction, namely the single exponential smoothing model, GM (Grey Model) model (1,1), and LV (Lotka-Vottera) model. GM and LV Model were used for predicting, decision making and conditional analysis. Mathematically, GM model could be used despite of its limitation on the data in which the model could process. This model has been developed and extended to Multiple Criteria Decision Making (MCDM) (Chiou, Tzeng & Cheng, 2004; Ji, Zou & Hu, 2010; Liu & Lin, 2010). GM model is a stochastic process in which its amplitude is varied in time based on generating series rather than on the raw one. GM Model is also developed using shooting and grey differential equation and needs less data, minimum of 4 periods of data. Liu & Lin (2010). Meanwhile, Lotka-Vottera Model is developed based on the different equations of the predator and the prey (Dang et al., 2016). It could be used for prediction with limited data and proven to be better in short-term forecasting (Hung, Tsai & Wu, 2014).

The forecast results of the three models showed that the single exponential smoothing had the lowest accuracy estimation, the GM model (1,1) had better accuracy and the LV Model had the best accuracy. Based on the value results from several measurements, the error of exponential smoothing model and GM (1,1) was greater than that of LV model. This means that the accuracy of the LV model was higher than the other two models. In general, the average precision level of the LV model was 89.7%, while the GM model (1,1) and exponential smoothing model were 86.36% and 65.94%, respectively. Therefore, in addition to the LV model, the GM model (1,1) can be an alternative for short-term forecasting with limited historical data. Thus, the exponential smoothing model was not suitable to be applied in this case. This study contributed a useful statistical tool that can be applied to time series data (Dang et al., 2016).

Exponential smoothing is a method of time series data forecasting that works based on the previous estimation and the percentage of forecast errors. The main problem of this technique is determining the optimal smoothing constant. In order to minimize forecasting errors, choosing an appropriate smoothing constant value is very important. In this study, a framework is developed for selecting the optimal value for the smoothing constant which minimizes the size of the forecast error such as the mean square error (MSE) and mean absolute deviation (MAD). Experiments to determine smoothing constant in this study were carried out by trial and error methods and the use of a non-linear method was proposed based on Excel Solver. In order to validate the proposed model, this study used time series data for demand for goods with monthly periods from 2010–2016. The most optimal smoothing constants using trial and error methods were 0.31 and 0.14 with MAD and MSE values of 6.0205 and 53.4287, respectively. While for non-linear methods, the optimal smoothing constants were 0.314 and 0.143 with MAD value of 6.0199 and MSE value of 53.4286. Although both methods gave similar results, the non-linear methods were much easier to use and required less time to obtain the optimal smoothing constant (Karmaker, 2017).

Hartomo, Subanar & Winarko (2016) conducted a research on rainfall forecasting using the exponential smoothing method. The research used monthly periods rainfall data from 2003 to 2014. They proposed a new method for finding smoothing constants using the Seasonal Planting Index (SPI) algorithm with index seasonal planting (*I*_*SP*_). Using *I*_*SP*_, the parameter of *α* was symbolized as *α*_*I*_*SP*__ which formulated as ${\alpha }_{{I}_{SP}}=1-\exp \left( -{I}_{SP} \right) $. Here, the exponential function was chosen to determine the smoothing value (*α*) since the smoothing value must be between 0 <*α* <1. The results of the rainfall data prediction test were obtained used SPI algorithm for RMSE value of 51.37, MAE value of 35.19, MSE value of 32.05, and MAPE value of 56.25 (Hartomo, Subanar & Winarko, 2016).

Recent research has successfully improved data time series forecasting accuracy using Fuzzy Type-2 time series. This time data series model used more observation in its forecast. The model was then combined with Particle Swarm Optimization (SPO) method. Combination between PSO and Type-2 Fuzzy model was to adjust the lengths of intervals in the universe of discourse that are employed in forecasting, without adding any interval numbers. The testing result showed the effectiveness and resilience of the proposed model compared to the fuzzy time series model and conventional time series model (Singh & Borah, 2014). Another relevant research showed the improvement of time series prediction accuracy using PSO hybrid fuzzy method. This method was used to predict the unknown future value proven to reduce the means squared error (RMSE). This also improves the accuracy as compared to the other models based on fuzzy time series (Huang, Hsieh & Lin, 2019).

A bit different from the previous research, there has been research on prediction model based on machine learning to improve the prediction accuracy of the conventional method. Machine learning -based prediction was performed using Terminated Hierarchical (ETH-CNN) to predict Hierarchical CU Partition Map (HCPM). The testing result showed that the coding complexity of High Efficiency Video Coding (HEVC) intra-mode could be drastically reduced by replacing the brute-force search with ETH-CNN. This approach exceeded the other sophisticated approaches in terms of reducing the HEVC complexity (Xu et al., 2018).

A research has been conducted on improving the HEV coding efficiency by optimizing neural network on Multiframe In-loop Filter (MIF). The research has demonstrated that the approach could improve the visual quality of each encoded frame by using the adjacent frames. The testing result revealed that the MIF approach has saved 1.621% of Bjøntegaard Delta Bit-Rate (BD-BR) on average. In other words, it significantly surpassed the filter in-loop standard with other cutting-edge approaches (Li et al., 2019). The development of machine learning-based prediction is carried out by adding the intrinsic feature of the prediction model. This research uses Python tools combined with web service to process and predict the data. The testing result demonstrates better prediction accuracy compared to standard machine learning models (He et al., 2020).

Therefore, improving prediction and classification method should be performed in a Deep Neural Networks (DNNs) environment on Computer Vision (CV) which are vulnerable to Adversarial Example (AEs). This research focuses on classification method by integrating three transformation with random coefficients well-adjusted according to the number of changes in the retained sample. Compared to the 4 advanced classification methods published in the Artificial Intelligence (AI) conference for the last two years, the proposed method shows an accuracy of more than 80% (Zeng et al., 2020).

A very recent research proposes the Ocean of Things (OoT) framework for monitoring the marine environment based on IoT (Internet of Thinks) technology. The OoT framework performs temperature predictions using a cloud model. The test results show that the framework obtain good prediction accuracy (Yang et al., 2020). A different prediction approach is used to address the limited resources of socially aware networks on online buying and selling cases using virtual currency. This research proposes an Equivalent-Exchange-based data forwarding Incentive Scheme (EEIS). This framework predicts the resource status of the two parties making transactions for optimization and efficiency of the network used. The test results show that the message delivery ratio has increased significantly and the EEIS framework can address the limitations of network resources (Xiong et al., 2020). Research with a different approach was carried out for scheduling efficiency in order to overcome bottlenecks in mmWave multi-Unmanned Aerial Vehicles (UAV) communications. The testing results have proved that prediction of transmission conditions and optimization of the proposed multi-UAV communication system scheduling algorithm are able to reduce the possibility of bottlenecks and increase the spectral efficiency of multi-UAV communication (Zhao et al., 2020).

Continuous development of artificial intelligence is increasing. Further research evaluates and warns the security risks of large-scale group activities based on the random forest algorithm. This research combines several model parameters from the random forest algorithm. Optimization experiments and random forest model training experiments are used for risk analysis with a classification accuracy of up to a maximum of 0.86. It can be concluded that the random forest algorithm has a good predictive ability in risk assessment in large-scale group activities (Chen et al., 2021). Another approach uses a semi-supervised prediction model, which utilizes an unsupervised clustering algorithm to form a fuzzy partition function. It then combines it with a neural network model to construct an information prediction function. The research results show that the proposed method produces better predictive accuracy than the conventional methods (Wen et al., 2021).

Another research combined the classical time series forecasting methods and machine learning methods. Starting with validating the methodology in combining the Bootstrap Aggregating (Bagging) with Exponential Smoothing method (Bergmeir, Hyndman & Benítez, 2016), this research used time series data for air freight demands which was further expanded with other time series data. After identifying previous researches on time series data forecasting in order to find aspects and problems, the new method i.e., Bagged Cluster ETS method was proposed because it uses the basic method of Bagging, Clusters and Exponential Refining.

### Single exponential smoothing

Single Exponential Smoothing (SES) model has been used by some researchers in previous studies for smoothing fluctuation in sequential demand patterns to provide stable estimations (Sopipan, 2015; Pagourtzi & Assimakopoulos, 2018). SES can be used for rainfall predictions (Wichitarapongsakun et al., 2016) using Eq. (1).

(1) \begin{eqnarray*}{F}_{t}={F}_{t-1}+\alpha ({X}_{t-1}-{F}_{t-1})=\alpha {X}_{t-1}+ \left( 1-\alpha \right) {F}_{t-1}\end{eqnarray*}

where *F*_*t*_ is the predicted rainfall at time *t*, *X*_*t*−1_ is the actual rainfall data at time *t* − 1 and *α* = [0,1] is the smoothing parameter constant, as well as, the significance or weight assigned to the data in time *t* − 1. If *α* is low, more weight will be given to the data in the past. If *α* is high, more weight will be given to the most recent data.

### Time series clustering

The method of identification and classification of large-scale time series data is done by grouping the time series data. This type of grouping differs from the grouping process for the cross-section data, especially in determining the distance technique for each cluster (Riyadi et al., 2017). The grouping on time series data requires a clustering algorithm or procedure to form clusters. If there is a set of unlabeled data objects, the choice of the correct clustering algorithm depends on the types of data available and the purpose of using the cluster. If the data to be clustered are the time series data, it can be analyzed whether the data have discrete or real values, whether data samples are uniform, whether they are univariate or multivariate, and whether data have the same length of series. Non-uniform sample data must be converted into uniform data before clustering operations can be performed. Grouping can be done using a variety of methods, from simple samplings based on the roughest sampling interval, up to sophisticated modelling and estimation approaches (Liao, 2005).

Various algorithms have been developed to classify different types of time series data. The aim of developing and modifying algorithms for static data grouping is that the time series data can be handled into static data so that the static data grouping algorithm can be used immediately (Chiou, Tzeng & Cheng, 2004). In general, the steps of grouping algorithm are described as follows.

Step 1: Starting with the initial cluster, denoted by *C*, it has a number of defined *k* clusters.

Step 2: For each time point, dissimilarity matrices are computed and all resultant matrices that have been calculated for all time points are saved for the calculation of trajectory similarity.

Step 3: In term of the generalized Ward criterion function, find cluster *C*′ that is better than cluster *C*. The cluster *C*′ is obtained from *C* by relocating one member of *C*_*p*_ to *C*_*q*_ or by swapping two members between *C*_*p*_ and *C*_*q*_, where *C*_*p*_, *C*_*q*_ ∈ *C*; *p*, q = 1 , 2, …, *k* and *p* ≠ *q*. If there are no such cluster, then stop; otherwise replace *C* with *C*′ and go back to Step 3.

This algorithm only works for time-series which have the same length because the distance between two time-series at some intersection is unclear (a point of time where one series has no value).

### k-means clustering algorithm

Clustering is a useful tool for data analysis. It is a method to find groups within data with the most similarity in the same cluster and the most dissimilarity between different clusters. One of the popular clustering algorithm is k-means algorithm (Macqueen, 1967).

Let *X* = {*x*_1_, …, *x*_*n*_} be a data in a *d*-dimensional Euclidean space *R*^*d*^. For a given 2 ≤ *c* ≤ *n*, *V* = {*v*_1_, …, *v*_*c*_} be the *c* cluster centers with Euclidean distance denoted by ∥*x*_*i*_ − *v*_*k*_ ∥ and $Z={ \left[ {z}_{ik} \right] }_{n\times c}$ be a partition matrix, where *z*_*ik*_ is the membership of data *x*_*i*_ ∈ *X*_*k*_ satisfying ${z}_{ik}\in \left\{ 0,1 \right\} $, ${\mathop{\sum }\nolimits }_{k=1}^{c}{z}_{ik}=1$, ∀*i*, ∀*k*. The k-means objective function can be written as,

(2) \begin{eqnarray*}J \left( Z,V \right) =\sum _{k=1}^{c}\sum _{i=1}^{n}{z}_{ik}\parallel {x}_{i}-{v}_{k}{\parallel }^{2}\end{eqnarray*}

where *z*_*ik*_ = 1 if *x*_*i*_ ∈ *X*_*k*_ and *z*_*ik*_ = 0 if *x*_*i*_⁄ ∈ *X*_*k*_.

The updating equations for memberships and cluster centers by minimizing $J \left( Z,V \right) $ are as follows,

(3) \begin{eqnarray*}{z}_{ik}& = \left\{ \begin{array}{@{}l@{}} \displaystyle 1 \end{array} \right. ,if\parallel {x}_{i}-{v}_{k}{\parallel }^{2}=\parallel {x}_{i}-{v}_{t}{\parallel }^{2}0,\text{otherwise}\end{eqnarray*}

(4) \begin{eqnarray*}{v}_{k}& = \frac{\sum _{i=1}^{n}{z}_{ik}{x}_{i}}{\sum _{i=1}^{n}{z}_{ik}} \end{eqnarray*}

The k-means clustering algorithm is described below.

Algorithm 1: k-Means Clustering

Input: data (*X*) and cluster number (*c*).

Given *ϵ* > 0 and ${v}^{ \left( 0 \right) }$. Let *t* = 1.

Step 1: Compute the membership ${z}^{ \left( t \right) }$ with *v*^(*t*−1)^ using (3).

Step 2: Update ${v}^{ \left( t \right) }$ with ${z}^{ \left( t \right) }$ using (4).

Step 3: Compare ${v}^{ \left( t \right) }$ and *v*^(*t*−1)^. IF $\parallel {v}^{ \left( t \right) }-{v}^{(t-1)}\parallel \lt \epsilon $, then STOP,

ELSE *t* = *t* + 1 and return to Step 1.

Output: clustered data (*W*_*k*_), *k* = 1, 2, …, *c*.

## The Proposed Method: Learning-based Single Exponential Smoothing Algorithm

As known in forecasting, Single Exponential Smoothing (SES) is used for data without trend or seasonal pattern. Meanwhile, Double Exponential Smoothing (DES) is used for trend data, and Triple Exponential Smoothing (TES) is used for seasonal data. Besides that, SES, DES, and TES need one (called alpha), two (called alpha and beta), and three (called alpha, beta, and gamma) parameters, respectively as their smoothing coefficients.

To simplify the seasonal pattern data, Hartomo, Subanar & Winarko (2016) proposed Exponential Smoothing Seasonal Planting Index (ESSPI) to group the data into three groups according to their seasonal planting term. There are three seasonal planting term in one year with four months long for each term, i.e., January-April, May-August, and September-December. The drawback of ESSPI is the grouping data have fixed terms for every year, even the seasonal planting period is changed for the coming years (Hartomo, Subanar & Winarko, 2016).

**Figure 1:**
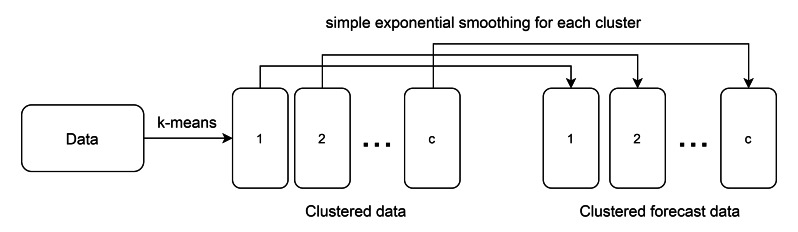
The idea of LSES algorithm.

To overcome the drawback of ESSPI, this paper uses the clustering algorithm to group data into seasonal clusters. Since the seasonal period can be changed every year (either the length of months or the grouped months), then k-means clustering algorithm is used to group the months with similar characterization. After k-means is applied, then SES is used to forecast each clustered data. In this case, it only needs one smoothing coefficient. Thus, in this paper, a modified single exponential smoothing, called Learning-based Single Exponential Smoothing (LSES) algorithm is proposed. Figure 1 shows the idea of LSES algorithm.

The existing literature suggests that in order to find the best smoothing value is by comparing the MSEs of different smoothing values. Smoothing value with the minimum MSE is chosen as the best smoothing value. This procedure is proven not to be effective. Therefore, this study provides a procedure to obtain the smoothing value by utilizing the clustering results.

Logically, smaller smoothing value is used for data with high changes. Meanwhile, higher smoothing value is used for data with low changes. The smoothing value that is closer to zero give higher smoothing effect than the smoothing value that is closer to one. The problem is how to determine the smoothing value. In this proposed method, the k-means clustering method is combined with the SES forecasting method. The clustering method is used to group the data with similar characteristics. These clustering results will be used to estimate the smoothing value. As known, the mean of data can be used as a point estimator of the whole data. Therefore, in this method, the mean of each cluster is used to estimate the smoothing value of each cluster. Since the mean of each cluster is vary, then the data normalization of each cluster is needed, in order to make the value of each cluster is in interval [0,1]. This normalization result can be used to determine the smoothing value, 0 < *α* < 1, directly. The procedure to find the smoothing value is described in Algorithm 2.

Algorithm 2: Procedure to find the smoothing value

Input: the clustered data (*W*_*k*_), *k* = 1, 2, …, *c*.

IF there is only one data in *W*_*k*_ or all the elements of *W*_*k*_ are 0, then *α*_*k*_ = *p*, where *p* is a constant, ELSE:

Step 1: For each cluster obtained from Algorithm 1, normalize each data in *W*_*k*_ using

(5) \begin{eqnarray*}{\hat {w}}_{k}= \frac{{w}_{k}-\min \nolimits ({W}_{k})}{\max \nolimits ({W}_{k})-\min \nolimits ({W}_{k})} \end{eqnarray*}

Step 2: Compute the smoothing value for each cluster (*α*_*k*_) using the average of the normalized clustered data, as follows,

(6) \begin{eqnarray*}{\alpha }_{k}=\text{mean} \left( {\hat {W}}_{k} \right) \end{eqnarray*}

Output: the smoothing value for each cluster (*α*_*k*_), *k* = 1, 2, …, *c*.

There are two computation steps in LSES, i.e., for the initialization and for the time period *t*. As written in Eq. (1), SES uses *X*_*t*−1_ and *F*_*t*−1_ to get *F*_*t*_, where *F*_1_ is assumed to be the same with *X*_0_ in the initialization process. In LSES, *F*_1_ is computed from the average of clustered data obtained from *X*_0_. Then, in time period *t*, LSES counts the forecast data *F*_*t*_ with the actual data *X*_*t*−1_. Furthermore, the smoothing values obtained from Algorithm 2 might be different for each iteration, depend on the clustered data formed in each iteration. The detailed LSES algorithm is presented in Algorithm 3.

Algorithm 3: LSES Algorithm

Input: actual data (*X*), number of clusters (*c*).

Step 1: For initialization period (*t* = 0, with actual data = *X*_0_ and forecast data = *F*_1_)

1. Group the actual data (*X*_0_) using k-means clustering algorithm in Algorithm 1 to obtain *W*_0,*k*_, *k* = 1, …, *c*.
2. For each cluster *k*, compute the forecasting data (*F*_0_) by computing the average of each cluster (${\overline{W}}_{0,k}$). All data in one cluster have the same forecasting data.
3. Find the smoothing coefficients for each cluster (*α*_*k*_) using Algorithm 2.
4. For each cluster *k*, compute the forecasting data (*F*_1_) with *α*_*k*_, *X*_0_, and *F*_0_ using (1), as follows. ${F}_{1}={\alpha }_{k}{X}_{0}+ \left( 1-{\alpha }_{k} \right) {F}_{0}$

Step 2: For the time period *t* (with actual data = *X*_*t*−1_ and forecast data = *F*_*t*_)

1. Group the actual data (*X*_*t*−1_) using k-means clustering algorithm in Algorithm 1 to obtain *W*_*t*−1,*k*_, *k* = 1, …, *c*.
2. Append *W*_*t*−1,*k*_ with *W*_*k*_. It means that if *t* = 1, then *W*_*k*_ contains of *W*_0,*k*_. If *t* = 2, then *W*_*k*_ contains of *W*_0,*k*_ and *W*_1,*k*_. If *t* = 3, then *W*_*k*_ contains of *W*_0,*k*_, *W*_1,*k*_ and *W*_2,*k*_, etc.
3. Find the smoothing coefficients for each cluster (*α*_*k*_) using Algorithm 2.
4. For each cluster *k*, compute the forecasting data (*F*_*t*_) with *α*_*k*_, *X*_*t*−1_, and *F*_*t*−1_ using Eq. (1), as follows, ${F}_{t}={\alpha }_{k}{X}_{t-1}+ \left( 1-{\alpha }_{k} \right) {F}_{t-1}$
5. Let *t* = *t* + 1 and go back to Step 2.1 until the prediction time *t* is reached.

Output: forecast data (*F*)

**Figure 2:**
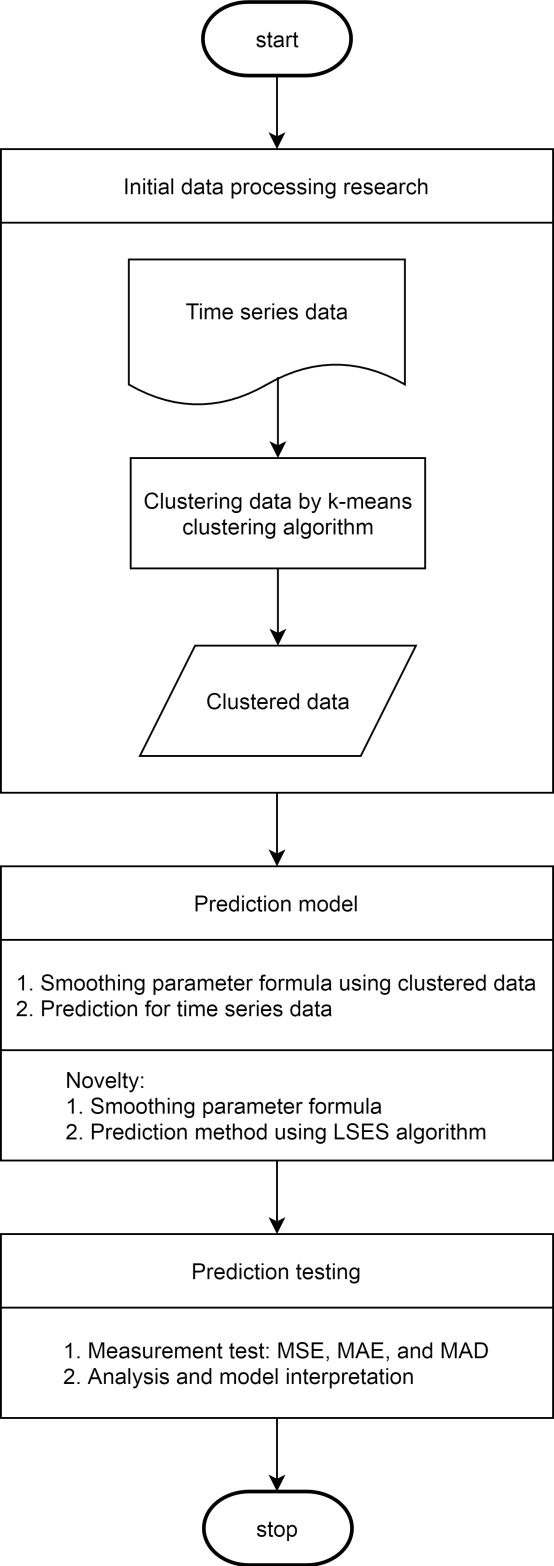
Flowchart of LSES algorithm.

For clear understanding, the flowchart for LSES algorithm is given in Fig. 2.

## Experimental Results

This section presents the experimental results for the rainfall data in Indonesia to show the performance of the proposed LSES algorithm. The rainfall data is obtained from Meteorology, Climatology, and Geophysical Agency (http://www.bmkg.go.id). This agency has the task to carry out governmental tasks in the fields of meteorology, climatology, air quality, and geophysics in accordance with applicable law and regulations. Indonesia has 34 provinces and one of them is Central Java. There are 23 climatology stations in Central Java. A climatology station records the rainfall data of one area in its scope. We use the rainfall data recorded by Adisumarmo climatology station for this experiment, starts from January 2007 until December 2019, as seen in Table 1.

**Table 1:** The rainfall data (in millimeter) from January 2007 until December 2019.

	Year												
Month	2007	2008	2009	2010	2011	2012	2013	2014	2015	2016	2017	2018	2019
Jan	390	503	176	561	347	752	215	284	677	566	498	492	521
Feb	419	183	300	318	313	477	510	474	440	295	340	297	339
Mar	548	206	278	383	268	232	532	625	104	341	514	242	462
Apr	244	167	56	195	120	273	331	174	155	432	444	444	198
May	114	65	42	92	20	183	94	143	265	406	241	241	200
Jun	101	1	25	21	147	0	37	17	277	138	101	101	222
Jul	50	0	0	0	101	0	11	0	0	101	76	0	186
Aug	0	0	0	0	8	0	0	0	0	130	0	0	12
Sep	29	0	2	7	76	0	0	1	3	244	34	0	6
Oct	348	1	24	28	85	0	2	311	69	235	80	80	135
Nov	246	120	139	211	178	159	125	360	182	156	340	220	196
Dec	181	105	328	212	483	489	394	169	262	183	283	366	487

According to the characteristic of annual rainfall data, the data can be divided into three categories, i.e., high, moderate, and low rainfall data, within one year (12 months). Thus, there are three clustered data (*X*_1_, *X*_2_, *X*_3_), with *c* = 3. For LSES algorithm, one constant is needed, i.e., *p*. In this annual rainfall prediction case, this constant can be calculated with *c*∕*n*_*c*_, where *c* is the number of clusters and *n*_*c*_ is the number of data in one cluster. In general, if 12 months are divided into three groups, equally, then one group has four months. Therefore, the constant *p* = 3∕4 = 0.75 is used in the computation.

The LSES algorithm is divided into two steps. Step 1 is started by grouping the rainfall data from January-December 2007 into three clusters, using k-means clustering algorithm. The average of each cluster is computed to obtain the forecast data of January-December 2007. It means that there is the same forecast data for months in the same cluster. After that, the forecast data for January-December 2008 are computed using (1) with the actual and forecast data of January-December 2007. Here, the smoothing value for each cluster is obtained from each clustered data (*W*_*k*_, *k* =1 , 2, …, 3) of January-December 2007, using Algorithm 2, therefore three smoothing values are obtained.

Step 2 is run first by grouping the data from January-December 2008 into three clusters. The corresponding clusters obtained from Step 1 and Step 2 are combined to be the clustered data (*W*_*k*_, *k* = 1 , 2, …, 3). Three clustered data are used to get the smoothing values for each cluster. Then, SES is used to forecast the data of January-December 2009. Step 2 is continued until the year to be predicted is reached, for this case is 2020.

**Figure 3:**
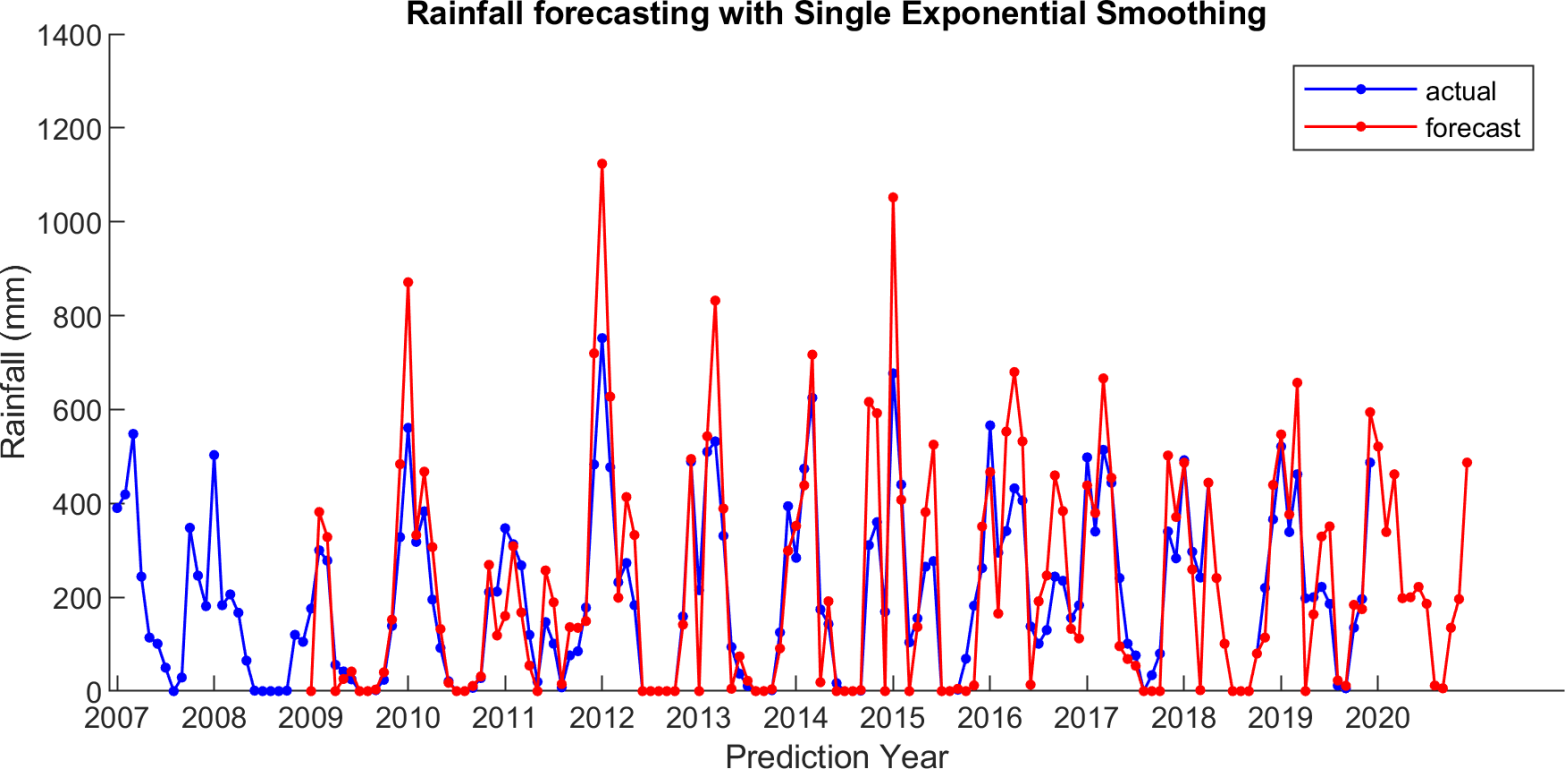
The actual and forecasting data for SES.

**Figure 4:**
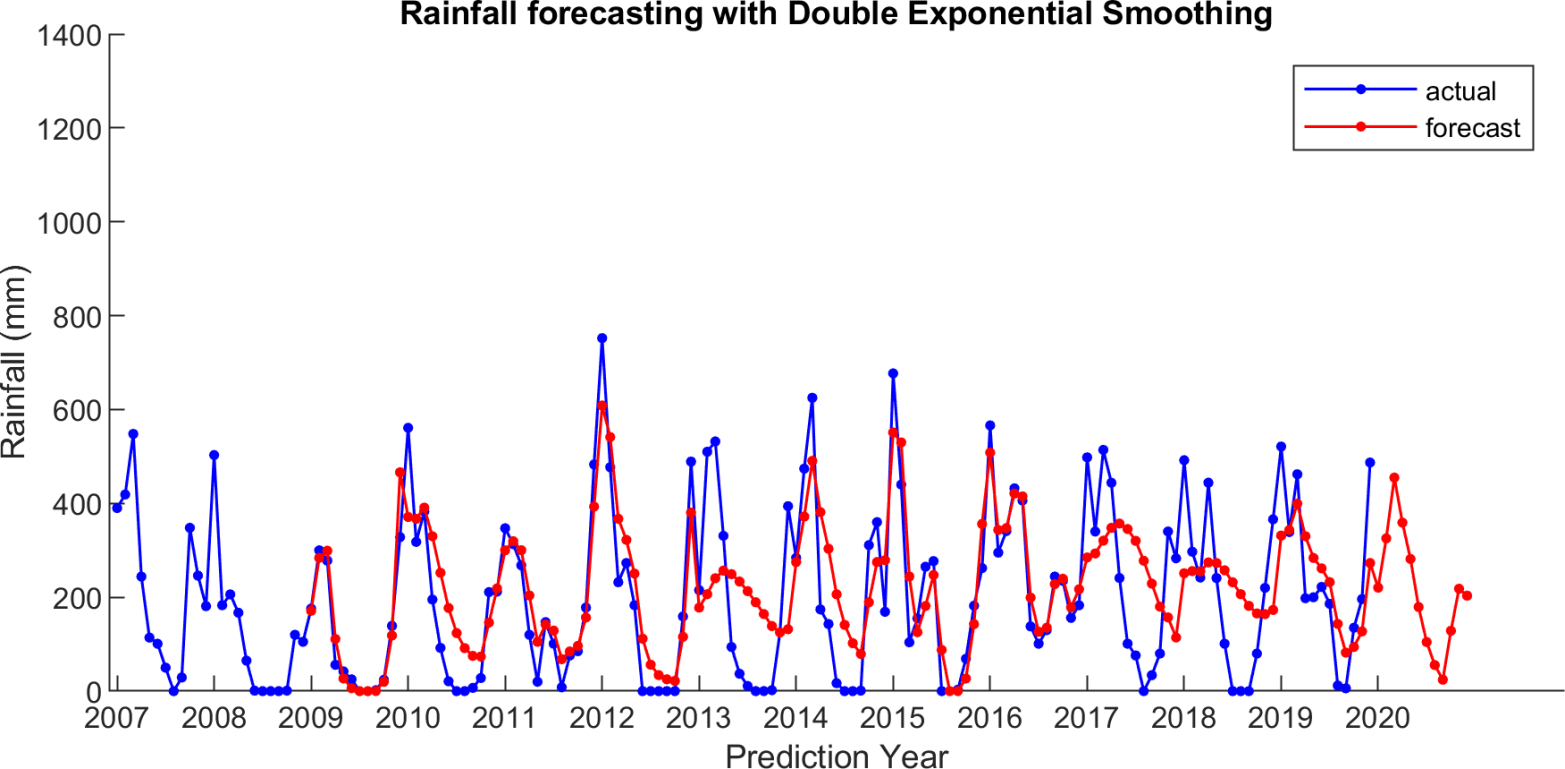
The actual and forecasting data for DES.

**Figure 5:**
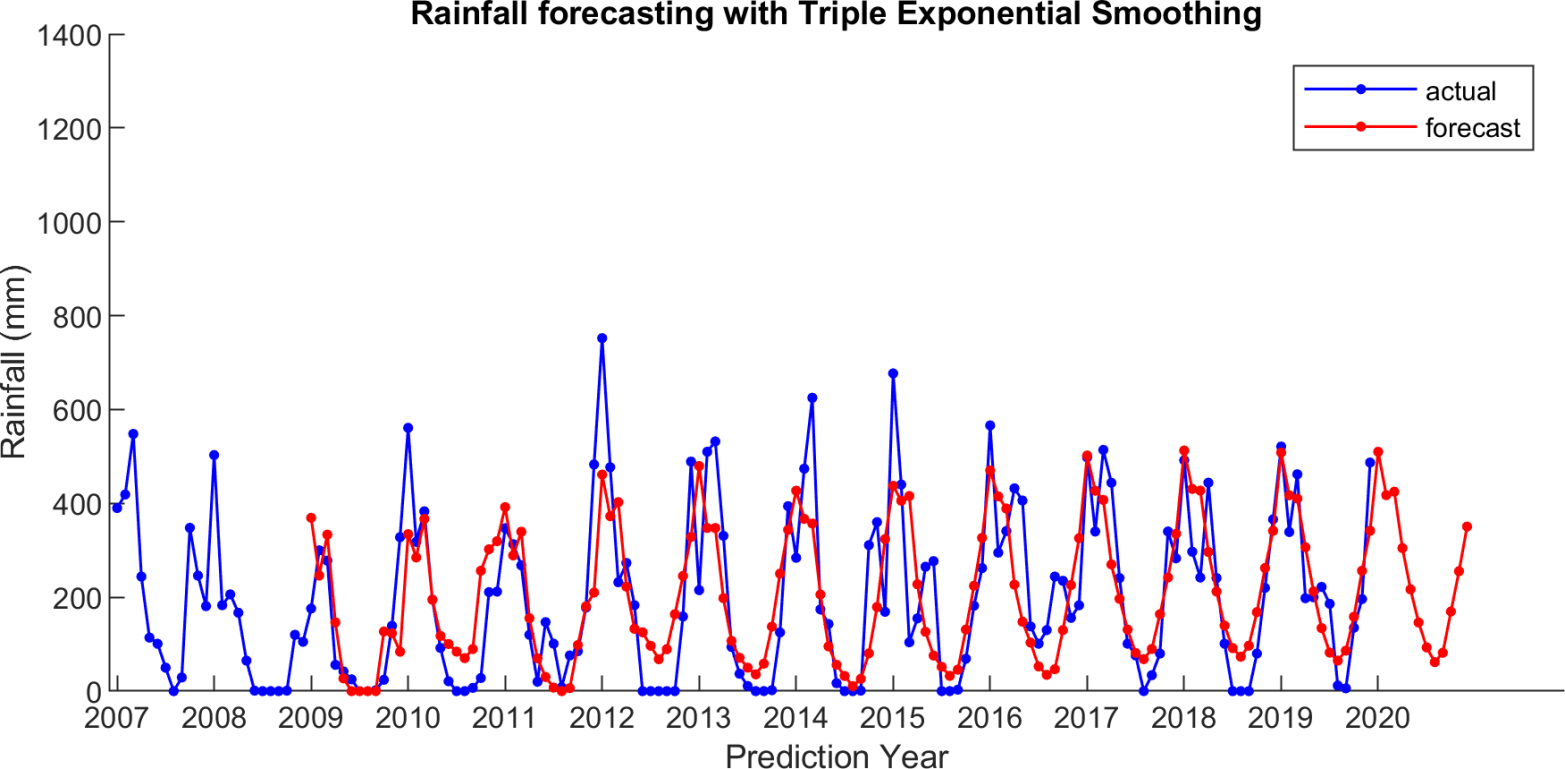
The actual and forecasting data for TES.

**Figure 6:**
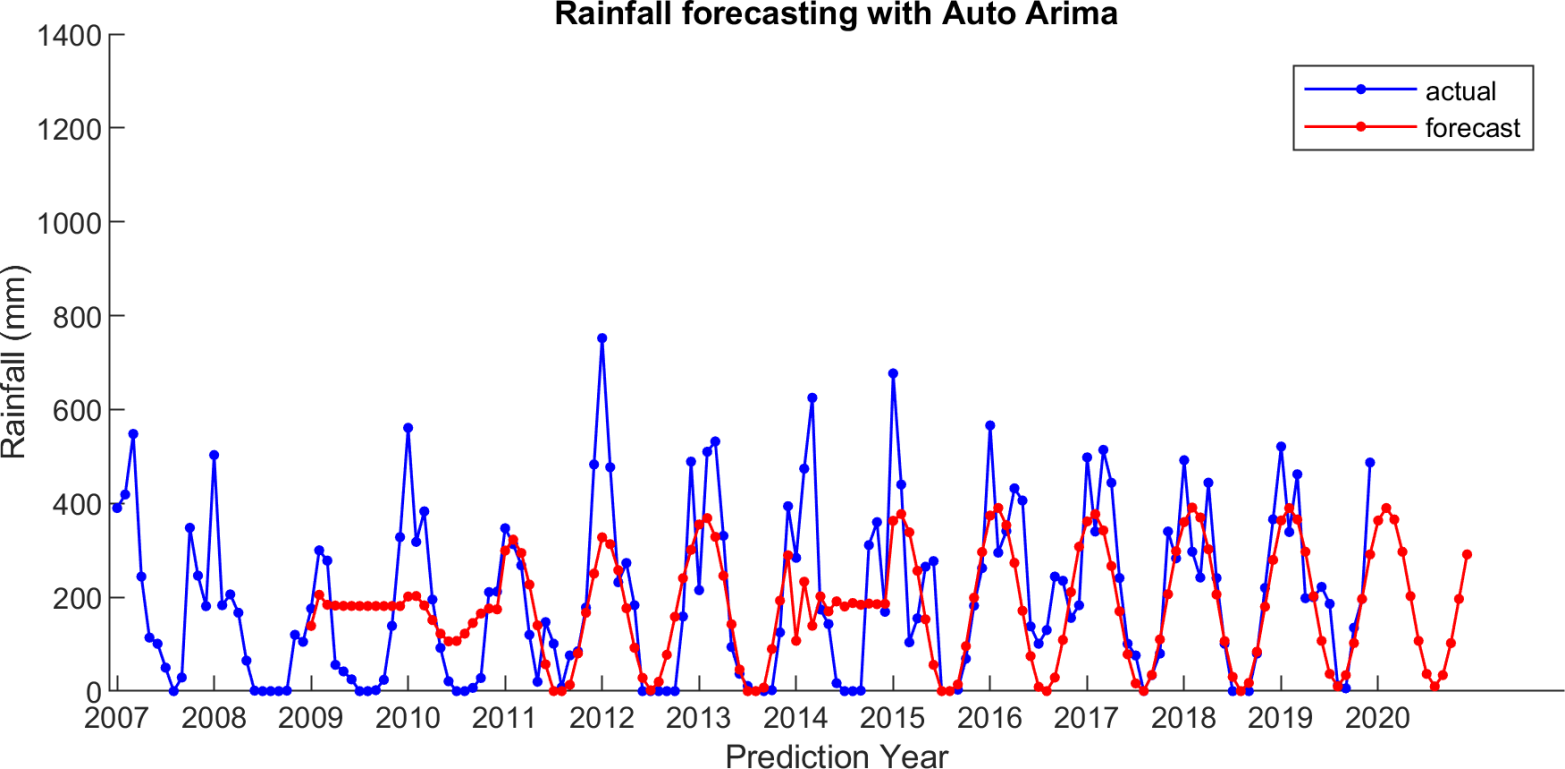
The actual and forecasting data for Auto Arima.

**Figure 7:**
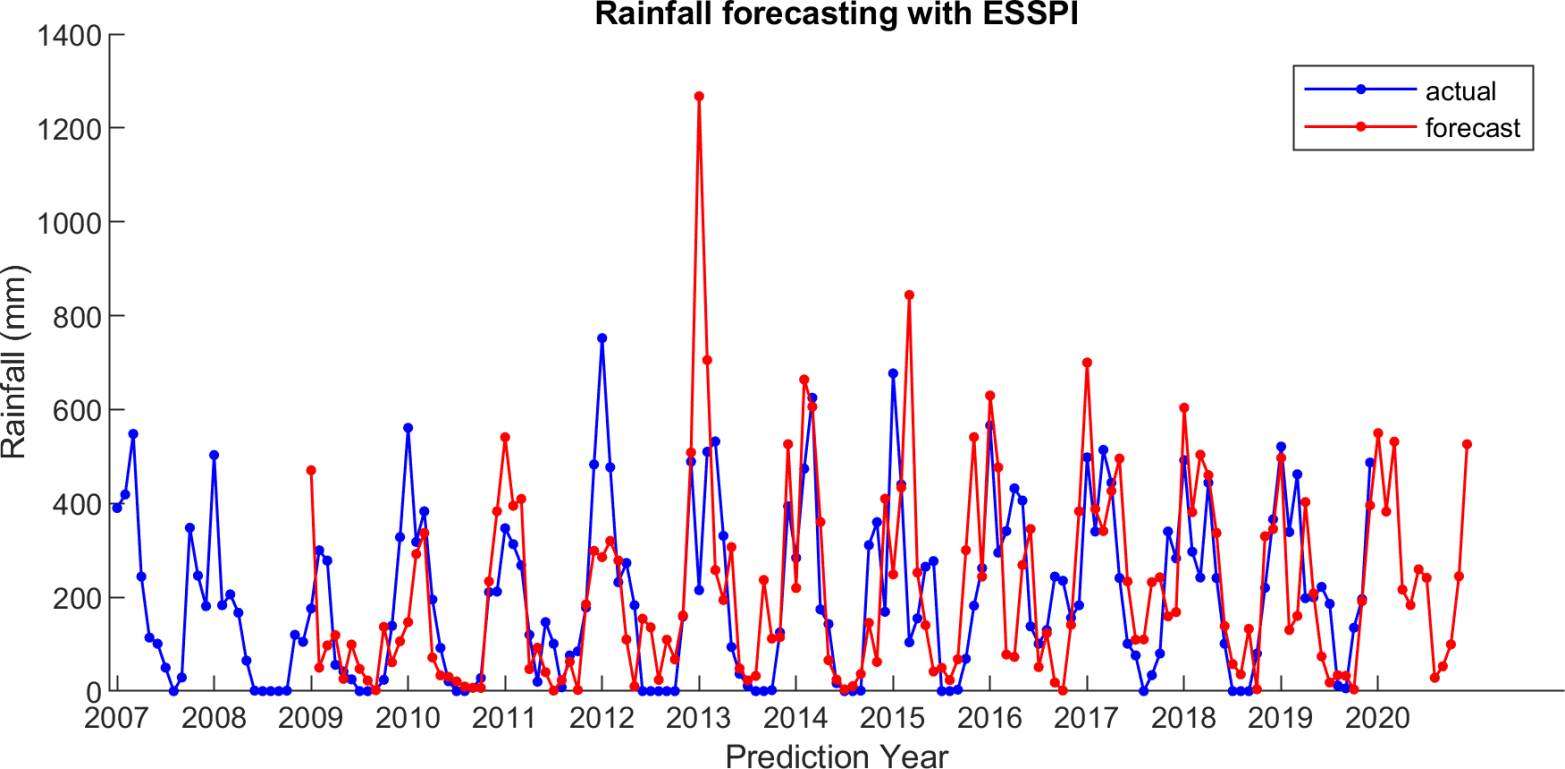
The actual and forecasting data for ESSPI.

**Figure 8:**
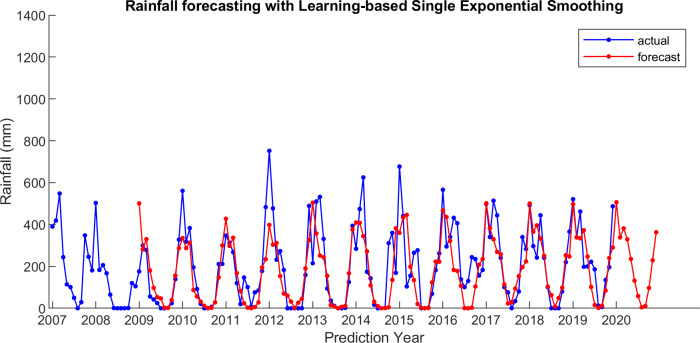
The actual and forecasting data for LSES.

For comparison, LSES algorithm is compared with five other algorithms, i.e., from SES, DES, TES, Auto Arima, and ESSPI. The line chart of actual and forecasting data for all periods of SES, DES, TES, Auto Arima, ESSPI, and LSES are depicted in Figs. 3–8, respectively. The actual data is from January 2007 until December 2019 and colored by blue color, while the forecast data is from January 2009 until December 2020 with red color. The *x*-axis is for prediction year and the *y*-axis is for rainfall prediction (in millimeter).

**Figure 9:**
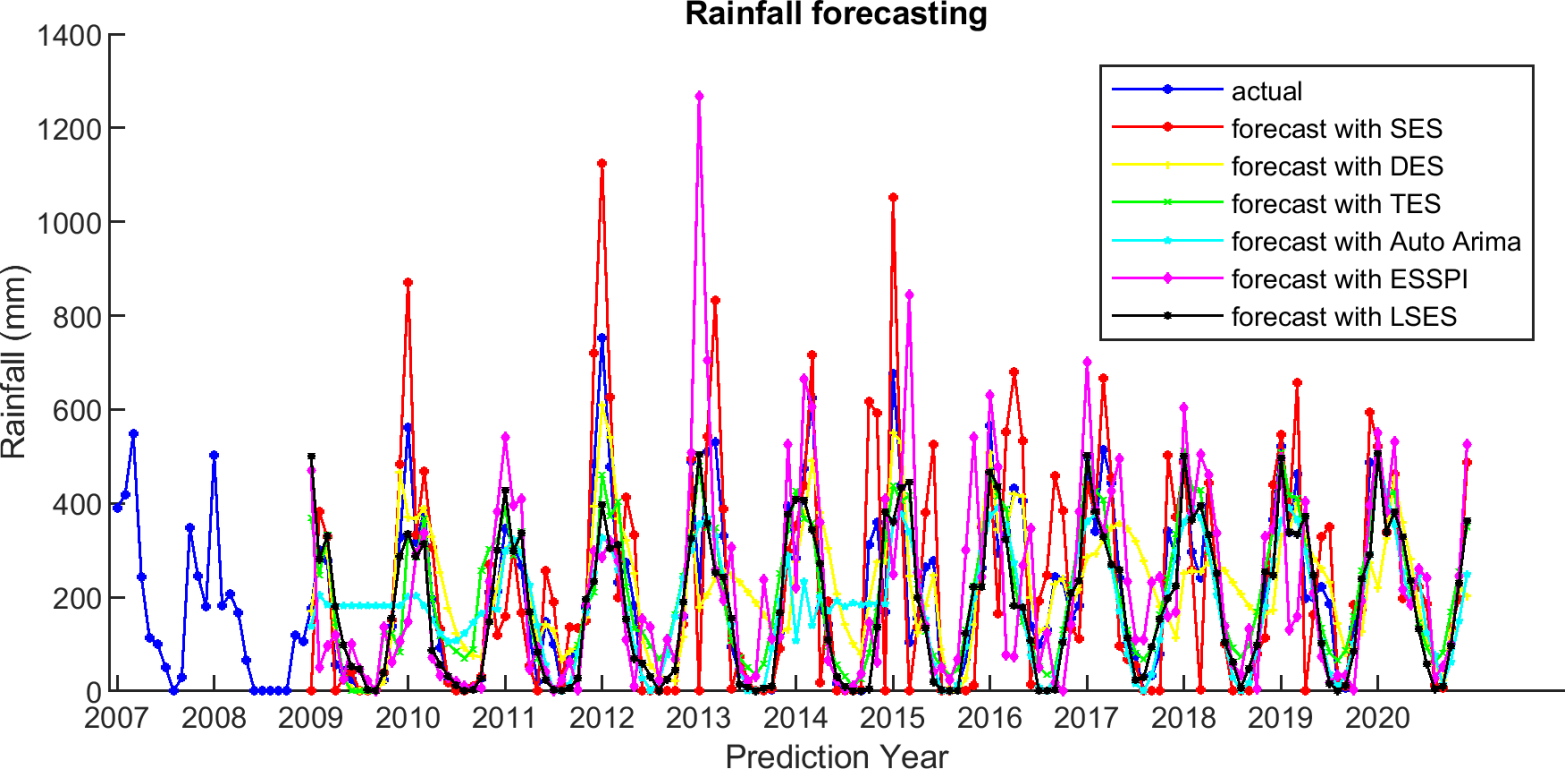
The actual and forecasting data for SES, DES, TES, Auto Arima, ESSPI, and LSES.

There are some smoothing parameters needed in SES, DES, TES, and Auto Arima. For SES, DES, TES, function in Python is used to get the best smoothing parameter values. For ESSPI, since there is no parameter needed, it follows the algorithm and applies to this data. Moreover, Fig. 9 shows the plot of the actual and forecasting data for all methods in one figure.

There are some parameters needed in SES, DES, TES, and Auto Arima. For SES, DES, TES, and Auto Arima, some functions in Python are used to get the best parameter values. The parameter values needed in SES, DES, TES, and Auto Arima are listed in Tables 2, 3, 4, and 5, respectively. While for ESSPI, since there is no parameter needed, the algorithm is followed and applied to this data.

**Table 2:** Parameter value for SES.

Year	Alpha for SES
2009	0.6975
2010	0.8055
2011	0.8734
2012	0.9178
2013	1.0000
2014	0.9878
2015	0.9541
2016	0.8947
2017	0.8805
2018	0.8823
2019	0.8866

**Table 3:** Parameter values for DES.

Year	Alpha for DES	Beta for DES
2009	0.8000	0.8000
2010	0.4000	0.0000
2011	0.6000	0.0000
2012	0.0000	0.0000
2013	0.1000	0.2000
2014	0.4000	0.0000
2015	0.7000	0.7000
2016	0.8000	0.0000
2017	0.1000	0.5000
2018	0.1000	0.1000
2019	0.4000	0.1000

**Table 4:** Parameter value for TES.

Year	Alpha for TES	Beta for TES	Gamma for TES
2009	0.0526	0.0526	0.4211
2010	0.2160	0.2155	0.0000
2011	0.2732	7.05E−73	1.62E−71
2012	0.2661	1.17E−55	1.79E−54
2013	0.2936	6.63E−90	4.27E−77
2014	0.1859	3.00E−51	1.51E−49
2015	0.1176	7.91E−35	4.75E−34
2016	1.43E−09	2.91E−31	1.46E−30
2017	8.24E−13	1.33E−79	6.65E−79
2018	4.24E−09	6.47E−44	8.05E−43
2019	6.9E−10	8.19E−84	4.09E−83

**Table 5:** Parameter model for Auto Arima.

Year	Parameter model for auto arima
2009	ARIMA(1,0,0)
2010	ARIMA(1,0,0)
2011	ARIMA(3,0,2)
2012	ARIMA(2,0,2)
2013	ARIMA(3,0,3)
2014	ARIMA(2,0,2)
2015	ARIMA(4,0,1)
2016	ARIMA(4,0,1)
2017	ARIMA(5,0,1)
2018	ARIMA(5,0,1)
2019	ARIMA(5,0,1)

Furthermore, for the forecasting accuracy, Mean Squared Error (MSE), Mean Absolute Error (MAE), Mean Absolute Deviation (MAD), and Mean Absolute Scaled Error (MASE) are computed to find the result performances. The average result of LSES algorithm from 100 experiments is compared with the results from SES, DES, TES, Auto Arima, and ESSPI. The formula for MSE, MAE, MAD, and MASE are $\mathrm{MSE}= \frac{{\mathop{\sum }\nolimits }_{t-1}^{n}{ \left( {F}_{t}-{X}_{t} \right) }^{2}}{n} $, $\mathrm{MAE}= \frac{{\mathop{\sum }\nolimits }_{t=1}^{n} \left\vert {F}_{t}-{X}_{t} \right\vert }{n} $, $\mathrm{MAD}= \frac{{\mathop{\sum }\nolimits }_{t=1}^{n} \left\vert {F}_{t}-{\overline{X}}_{t} \right\vert }{n} $, and $\text{MASE}= \frac{ \left\vert {X}_{t}-{F}_{t} \right\vert }{ \frac{1}{n-1} \left( {\mathop{\sum }\nolimits }_{i=2}^{n} \left\vert {X}_{t}-{X}_{t-1} \right\vert \right) } $respectively, where *F* is the forecasting data, *X* is the actual data, *t* is time period, and *n* is number of time period.

**Figure 10:**
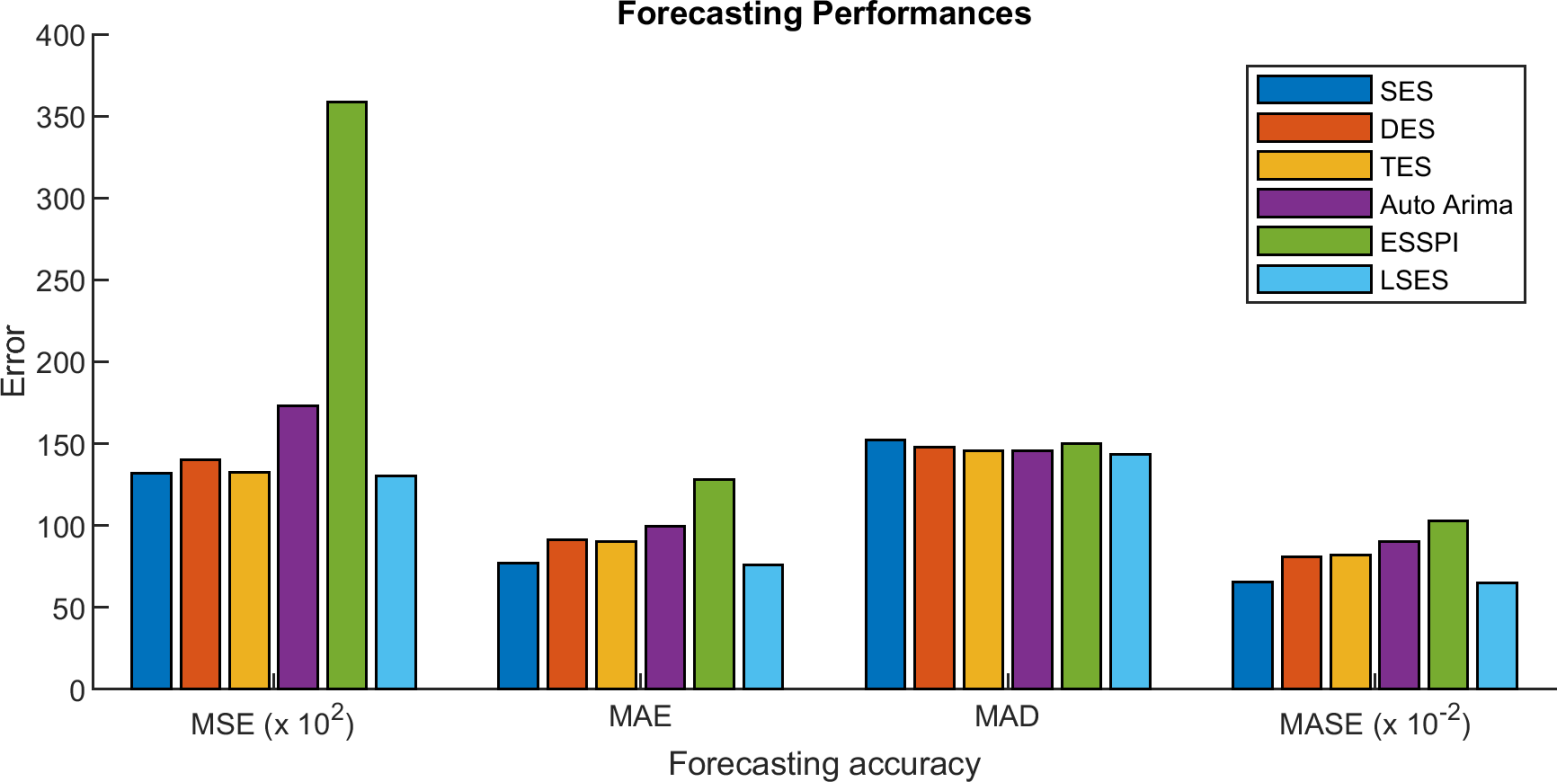
Comparisons of MSE, MAE, MAD, and MASE in the form of graphs.

The comparison results of MSE, MAE, MAD, and MASE are given in Tables 6, 7, 8, and 9 respectively. Figure 10 shows the error values in the form of graphs. The averages of MSE, MAE, MAD, and MASE for 11 prediction years from 2009 until 2019 are compared. From those tables, SES gives 13,212, 77.31, 152.23, 0.654; DES gives 14,032.78, 91.12, 147.9, 0.809; TES gives 13,246.39, 90.32, 145.76, 0.818; Auto Arima gives 17,287.5, 99.73, 145.69, 0.901; ESSPI gives 35,866.34, 128.15, 152.06, 1.030; and LSES gives 13,007.91, 75.87, 143.34, 0.648 for average of MSE, MAE, MAD, and MASE, respectively. Thus, LSES obtains the smallest averages of MSE, MAE, MAD, and MASE compared with other algorithms, i.e., SES, DES, TES, Auto Arima, and ESSPI. It means that LSES provides a promising algorithm in forecasting.

**Table 6:** The comparisons of MSE for SES, DES, TES, Auto Arima, ESSPI, and LSES.

Prediction year	SES	DES	TES	Auto Arima	ESSPI	LSES
2009	5705.64	1989.76	10232.94	18750.42	21869.92	10812
2010	10819.15	11792.50	12545.17	21651.31	18647.78	5177
2011	11053.52	2552.99	9456.64	8784.58	11459.18	7495
2012	17028.64	6800.60	18938.27	24943.95	30061.03	17010
2013	13356.17	36473.84	16022.76	9504.77	114328.43	14346
2014	18055.03	16974.25	18354.29	42057.01	21399.73	22574
2015	22447.24	6019.21	19684.06	19296.19	83469.61	24391
2016	21688.82	1022.12	18542.51	19710.83	37136.14	20413
2017	7723.72	34515.61	6603.73	9087.56	23357.79	7084
2018	6303.07	24281.10	9172.22	6196.12	11618.93	3124
2019	11151.01	11938.61	6157.68	10179.73	21181.20	10661
Average of MSE	13212.00	14032.78	13246.39	17287.50	35866.34	**13007.91**

**Table 7:** The comparisons of MAE for SES, DES, TES, Auto Arima, ESSPI, and LSES.

Prediction Year	SES	DES	TES	Auto Arima	ESSPI	LSES
2009	48.57	24.63	66.46	128.02	113.42	60.0491
2010	60.28	91.60	87.12	117.66	76.90	43.4652
2011	79.84	39.83	66.89	68.37	89.31	64.3982
2012	72.35	71.73	121.36	112.98	126.54	93.9574
2013	72.83	167.70	102.99	75.50	201.34	75.8877
2014	93.62	120.05	105.93	166.65	108.18	99.9427
2015	101.96	63.75	107.86	94.56	198.20	98.8299
2016	133.69	25.07	118.39	124.96	161.95	119.0163
2017	68.86	173.20	67.42	71.31	135.57	58.3338
2018	38.53	133.98	80.93	59.57	86.66	42.4869
2019	79.82	90.76	68.12	77.43	111.58	78.2242
Average of MAE	77.31	91.12	90.32	99.73	128.15	**75.87**

**Table 8:** The comparisons of MAD for SES, DES, TES, Auto Arima, ESSPI, and LSES.

Prediction Year	SES	DES	TES	Auto Arima	ESSPI	LSES
2009	109.57	110.21	109.56	126.88	61.28	114.90
2010	147.92	150.38	146.84	144.33	72.52	144.33
2011	120.00	120.16	110.70	111.72	106.54	110.50
2012	211.43	196.82	194.37	187.25	144.33	187.25
2013	174.13	175.06	176.45	171.44	115.13	170.56
2014	169.84	172.14	159.18	159.05	187.25	158.17
2015	157.20	151.96	152.06	147.67	213.15	147.67
2016	131.26	117.72	115.25	118.94	165.39	118.44
2017	157.25	157.25	157.25	166.00	162.30	159.18
2018	146.65	140.08	144.38	144.44	114.94	140.08
2019	149.24	135.09	137.25	124.83	160.17	125.70
Average of MAD	152.23	147.90	145.76	145.69	152.06	**143.34**

**Table 9:** The comparisons of MASE for SES, DES, TES, Auto Arima, ESSPI, and LSES.

Prediction Year	SES	DES	TES	Auto Arima	ESSPI	LSES
2009	0.54	0.39	0.80	1.99	1.42	0.53
2010	0.46	1.01	0.91	1.17	0.56	0.34
2011	0.72	0.40	0.71	0.72	0.82	0.68
2012	0.38	0.54	0.88	0.70	0.80	0.59
2013	0.53	1.59	0.78	0.53	0.62	0.51
2014	0.70	0.94	0.74	1.20	0.81	0.76
2015	0.66	0.50	0.82	0.64	1.52	0.68
2016	1.51	0.24	1.33	1.31	1.89	1.36
2017	0.62	1.50	0.65	0.58	1.15	0.56
2018	0.36	1.08	0.75	0.46	0.74	0.39
2019	0.72	0.70	0.62	0.60	1.02	0.71
Average of MAE	0.654	0.809	0.818	0.901	1.030	**0.648**

**Table 10:** CoV of LSES.

	High rainfall data	Moderate rainfall data	Low rainfall data
Standard deviation	83.60	32.65	30.81
Mean	336.31	149.52	28.13
**Coefficient of variation**	**0.25**	**0.22**	**1.10**

Moreover, coefficient of variation (CoV) is used to find the forecast stability, where $\mathrm{CoV }= \frac{\sigma }{\mu } $ with *σ* is the standard deviation and µis the average (mean). Smaller values of a CoV indicates stability, since the variability of the data around their mean is small. In the experiments, the rainfall data are divided into three groups with LSES, i.e., high, moderate, and low rainfall data, so the CoV is computed according to those groups.

As seen from Table 10, the results of CoV for high, moderate, and low rainfall data are about 0.22, 0.25, and 1.10, respectively, which means their variations are small. Therefore, LSES is stable and can be used for forecasting data.

Since LSES obtains the best performance, therefore, LSES algorithm is used to predict the rainfall in 2020 and compare it to the actual data of 2020. The result is shown in Table 11. Moreover, the predictions obtained from SES, DES, TES, Auto Arima, and ESSPI are also given in this table for comparison. As can be seen from this table, LSES produces MSE of 1716.39, smaller than MSEs from other methods.

**Table 11:** Rainfall prediction for 2020.

Month	Actual	SES		DES		TES		AA		ESSPI		LSES	
		Forecast	MSE	Forecast	MSE	Forecast	MSE	Forecast	MSE	Forecast	MSE	Forecast	MSE
Jan	451	521.00	4900.00	220.40	53174.63	509.99	3479.87	501.49	2549.73	549.67	9735.92	498.36	2242.97
Feb	367	339.00	784.00	325.51	1721.50	417.51	2550.93	423.40	3181.00	382.40	237.12	329.71	1390.54
Mar	296	462.00	27556.00	454.91	25253.00	424.88	16609.07	354.84	3462.41	531.53	55475.48	340.48	1978.47
Apr	248	198.00	2500.00	358.96	12311.49	304.78	3223.82	255.02	49.32	216.25	1007.79	283.68	1273.06
May	226	200.00	676.00	281.59	3089.80	216.86	83.48	190.21	1281.00	183.15	1835.94	224.47	2.34
Jun	83	222.00	19321.00	179.22	9258.26	146.29	4005.62	248.75	27474.47	259.49	31148.01	129.73	2183.69
Jul	41	186.00	21025.00	104.51	4033.70	92.96	2699.67	125.17	7084.47	241.31	40124.53	53.56	157.75
Aug	2	12.00	100.00	55.51	2862.88	61.75	3570.21	76.42	5537.98	28.51	702.82	3.25	1.56
Sep	43	6.00	1369.00	24.28	350.35	81.82	1506.77	15.01	783.64	52.92	98.40	8.67	1178.55
Oct	124	135.00	121.00	128.62	21.32	169.50	2070.24	62.67	3761.72	99.54	598.20	92.37	1000.46
Nov	249	196.00	2809.00	218.11	954.01	255.43	41.32	150.29	9744.28	244.62	19.19	214.72	1175.12
Dec	273	487.00	45796.00	203.31	4856.58	350.57	6016.82	248.00	625.18	526.05	64033.66	362.51	8012.04
Average			10579.75		9823.96		3821.48		5461.27		17084.75		**1716.38**

Furthermore, the experiment is extended to better reflect the value of the presented network intrusion detection model. LSES is used to investigate the applicability of the model through a real case study of intrusion detection system. The data is obtained from Canadian Institute for Cybersecurity (https://www.unb.ca/cic/datasets/). In this data, a two-layered approach is used to generate benign and darknet traffic constitutes Audio-Stream, Browsing, Chat, Email, P2P, Transfer, Video-Stream, and VOIP which is generated at the second layer. Intrusion detection can be analyzed and identified visually by three features, i.e., average packet size, total length of forward packets, and total length of backward packets. This experiment uses four attributes, i.e., src_port (source port), dst_port (destination port), timestamp, and total_fwd_packet (total of forward packet), where the total of forward packet is being predicted (Lopez, 2019). Data with a unique combination of src_port, dst_port, and timestamp are chosen.

**Figure 11:**
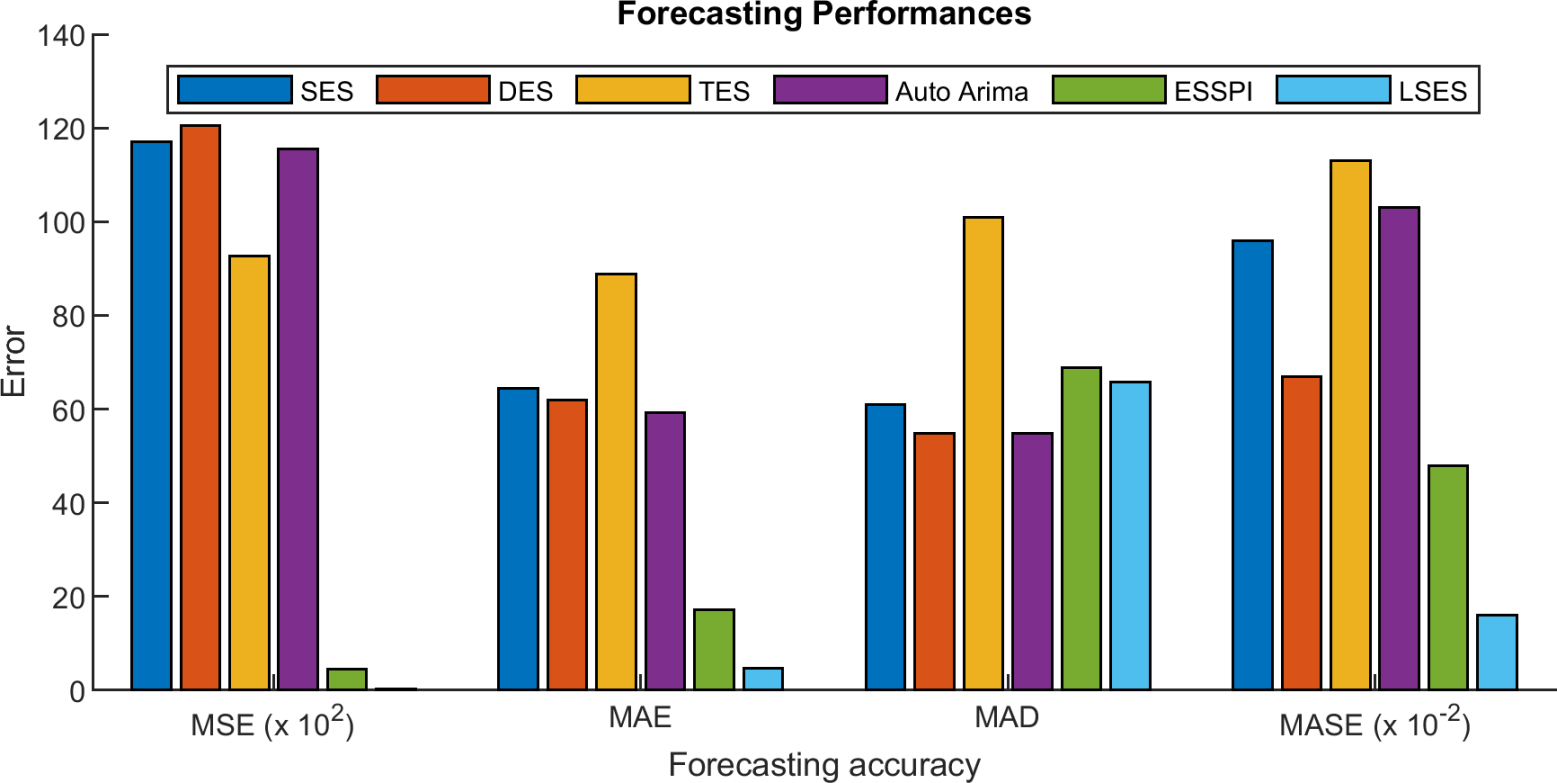
Forecasting performances of intrusion detection prediction.

Table 12 shows the prediction of intrusion detection with six methods. Since LSES uses clustering results for the prediction, then the result of LSES can detect which ports have high and low values of total forward packet. Table 13 is the MSE, MAE, MAD, and MASE of SES, DES, TES, Auto Arima, ESSPI, and LSES. From Table 13, LSES gives the smallest MSE, MAE, and MASE, while for MAD, since LSES works with clustering method and MAD uses the average of all forecasting data, then the MASE for LSES cannot obtain the smallest one. Figure 11 expresses the error values in the form of graphs.

**Table 12:** Intrusion detection predictions.

Total fwd packet	SES	DES	TES	Auto Arima	ESSPI	LSES
250	273.58	488.00	289.26	271.91	265.55	252.42
270	484.94	247.00	174.57	243.56	256.43	264.07
264	250.39	248.10	183.20	275.18	234.66	260.58
258	274.65	263.70	173.90	271.50	256.79	257.08
265	268.09	268.74	162.54	272.42	256.00	261.16
260	268.00	262.35	162.91	272.42	244.17	258.25
236	261.10	256.12	234.24	273.34	219.80	244.27
263	256.07	266.20	119.56	274.00	222.86	259.99
244	267.83	257.87	128.18	272.42	239.33	248.93
248	256.17	244.72	118.89	274.00	261.99	251.26
37	244.17	223.02	107.52	275.57	44.09	28.00
1	222.32	228.93	107.89	278.46	40.79	10.00

**Table 13:** Intrusion detection prediction errors.

Forecast method	MSE	MAE	MAD	MASE
SES	11708.86	64.37	60.944	0.96
DES	12054.28	61.92	**54.895**	0.67
TES	9269.64	88.89	100.963	1.13
Auto Arima	11549.40	59.30	54.898	1.03
ESSPI	445.33	17.20	68.752	0.48
LSES	**28.81**	**4.65**	65.778	**0.16**

## Conclusions

To sum up with, the paper proposed the learning-based single exponential smoothing (LSES) forecasting algorithm. By using k-means clustering algorithm and single exponential smoothing, LSES produce good forecasting results. This algorithm groups the data in the past by using k-means clustering algorithm, according to their characteristics. Since single exponential smoothing needs one smoothing parameter value, LSES computes this smoothing value with the clustering result by learning-based procedure, automatically. Experimental result and comparisons demonstrate the effectiveness of the proposed LSES algorithm to obtain the prediction data in the future. It has the smallest mean squared error of 13,007.91 and the average improvement rate of 19.83%. For future research, since there is still a certain gap between the actual and forecast data of LSES, it would be better if some deep learning methods, such as MLP (Multilayer Perceptron), CNN (Convolutional Neural Network), or LSTM (Long-Short Term Memory Network) are used to automatically learn the temporal dependencies and handling the temporal structures, like trends or seasonality.

## Additional Information and Declarations

### Data Availability

The following information was supplied regarding data availability: Code and raw data for LSES is available at https://github.com/yessno24/lses/tree/main.
